# Health surveillance for SARS-CoV-2: infection spread and vaccination coverage in the schools of Modena province, Italy

**DOI:** 10.3389/fpubh.2023.1240315

**Published:** 2023-10-27

**Authors:** Stefania Paduano, Maria Chiara Facchini, Lucia Borsari, Alessandra D’Alterio, Laura Iacuzio, Antonella Greco, Elisabetta Fioretti, Giacomo Creola, Zaynalabedin Kahfian, Stefano Zona, Annalisa Bargellini, Tommaso Filippini

**Affiliations:** ^1^Department of Biomedical, Metabolic and Neural Sciences – Section of Public Health, University of Modena and Reggio Emilia, Modena, Italy; ^2^Department of Public Health – Public Hygiene Service, Local Health Authority of Modena, Modena, Italy; ^3^Infection Control Strategic Group, Local Health Authority of Modena, Modena, Italy; ^4^School of Public Health, University of California, Berkeley, Berkeley, CA, United States

**Keywords:** SARS-CoV-2, vaccination, schools, infection spread, health surveillance

## Abstract

**Introduction:**

In Italy, over 4.8 million individuals aged 0–19 years have been infected with SARS-CoV-2. This study aims to evaluate the spread of SARS-CoV-2 within schools in Modena province and the influence of anti-SARS-CoV-2 vaccination coverage.

**Methods:**

We performed a survey in the period 1 September-15 December 2021, involving student population aged 0–19 years and related teachers screened for SARS-CoV-2 infection using nasopharyngeal swab after the detection of an index case within their class. During the study period, vaccination against SARS-CoV-2 was actively offered to all subjects aged ≥12 years.

**Results:**

A total of 13,934 subjects were tested, 12,534 students and 1,400 teachers (594 classes). We identified a total of 594 and 779 index and secondary cases, respectively. We found that 9.8% of students and 10.6% of teachers were positive for SARS-CoV-2. Overall at the test time, 32.5% were vaccinated with at least one dose of anti-SARS-CoV-2 vaccine. Among secondary cases, 7.8% were vaccinated compared to 34.9% among negative tested subjects. A higher secondary attack rate was for non-vaccinated subjects rather than vaccinated ones (8.1% vs. 1.4%). Higher secondary attack rates were reported for subjects attending infant and primary school (5.9 and 9.6%, respectively). Lower secondary attack rates were for those who attended middle school (4.9%) and especially high school (1.7%).

**Conclusion:**

Our results highlight the differential spread of the infection within various educational settings and that the vaccination, available in the study period for the population aged ≥12, have mitigated SARS-CoV-2 spread in high and middle schools.

## 1. Introduction

In Italy, over 4.8 million individuals aged 0–19 years have been infected by SARS-CoV-2 virus, representing about 19% of all reported cases since the beginning of the pandemic (1, 2). Schools closures, home confinement, and social distancing measures have disrupted the children daily routines and adolescents and limited their access to social activities, which can have negative effects on their mental health and well-being, with a significant decrease in quality of life (3–5). COVID-19 has strictly related to children and adolescents’ development due to confinement measures (6).

Worldwide, one of the main preventive strategy against the COVID-19 pandemic was schools closure, even if, in the early stages of the pandemic, the role of children in the transmission of the virus was unknown (7). In Italy, most of the children did not present symptoms and for this reason they were quarantined at home without any molecular or antigenic tests (8). Additionally, physical distancing and specifically distance learning in school setting have been implemented in several countries, including Italy; as a consequence, no increased risk of infection was reported among workers of the education sector (9, 10).

Keeping school as a safe and accessible environment is of utmost importance and goes beyond the primary objective of the educational needs as it affects the social and mental development of children (6), and it is the primary means to reduce inequality (11). The role of school in SARS-CoV-2 spread and the effectiveness of its closure in the control of the epidemic has been long debated. Several studies showed that the prevalence of positive cases in schools is lower than in the general population when appropriate mitigation measures are implemented, as well as the number and size of clusters in educational settings are generally smaller (12–16).

The health behavioral policies adopted before the availability of the vaccination and still in use helped in mitigating the risk of viral spread in schools; particularly, contact tracing turned out to be useful to promptly isolate infected students and staff (17). Regular testing could be also a key strategy to control the epidemic in school settings characterized by lower vaccination coverage compared to the general adult population or after the waning of vaccine protection, minimizing lost days (18). A modeling study in simulated elementary and middle schools found that screening tests eased in-person schooling with limited transmission risk, and test-to-stay policies were associated with increased school attendance and only little incremental transmission. Epidemiological surveillance has been identified as a useful, low-cost option for the detection of outbreaks and identification of school environments that could benefit from increased mitigation (19).

A widespread increase in vaccination coverage for the pediatric population has been strongly recommended (20, 21). Initially, the European Medicines Agency (EMA) recommended the administration of the vaccine to children aged 12 years and above. Subsequently, this recommendation was extended to encompass children below 12 years (22). After that, on 7 December 2021, the Italian Ministry of Health extended the use of the vaccine to children aged 5–11 years (23). In Emilia Romagna Region, since 16 December 2021 vaccination against SARS-CoV-2 has been also available for subjects aged 5–11 years (24).

The acceptance of vaccination against SARS-CoV-2 among parents has a significant role: the safety of vaccination is considered the most important factor affecting vaccine hesitancy during childhood (25). Therefore, advocating the safety and efficacy of vaccines through trusted and institutional sources might help the development of a sense of confidence and security among parents and the general public (26, 27). Researchers underlined that virus circulation among students, educators, staff, and their family members is high when a highly infectious variant predominates in unvaccinated students. Nevertheless, the implementation of mitigation measures or use of vaccinations in students can substantially reduce these modeled risks (28). Especially, vaccination remains the most effective and sustainable strategy for risk reduction, thus efforts should focus on the increase of coverage and use of booster doses among eligible students and school staff (29).

Since children infected with SARS-CoV-2 are mostly without symptoms or with mild non-specific symptoms, outbreaks are difficult to record in educational settings (30, 31). Therefore, this study aims to assess the spread of SARS-CoV-2 within schools in Modena province and the influence of vaccination coverage in these settings.

## 2. Methods

This study was approved by the “Area Vasta Emilia Nord” Ethics Committee (approval no. AUO/0017667/20 of June 25, 2020).

### 2.1. Study population

We performed a survey in school settings of Modena province (Northern Italy) in the period from September 1 to December 15, 2021. We considered all students and teachers who were screened for SARS-CoV-2 infection through nasopharyngeal swab after the detection of an index case within their classroom, and the related onset of secondary cases.

According to ministerial and regional policies (32), teachers and students were tested if they attended the same class with a confirmed positive case in the 48 h prior test or symptoms onset (school contact). Differently from the general definition of close contact, the distance from the index case was not considered to define a school contact who has to be included in the screening. Screening tests were performed with molecular or antigenic tests, but in case of positive result with antigenic test, individuals needed molecular test to be considered a confirmed secondary case. Indication for quarantine varied from nursery, infant, primary and secondary (middle and high) schools, but the execution of at least one nasopharyngeal swab for SARS-CoV2 was mandatory for the re-admission to all the grades of school. Non-adherence to screening tests was very low (<5%). In the analysis, these few subjects were considered negative for SARS-CoV-2 screening. We considered as a school cluster the presence of 2 or more SARS-CoV-2 cases (students or teachers with a positive molecular SARS-CoV-2 test, regardless the occurrence of correlated symptoms) attending the same classroom within a period of 14 days. In the considered period, vaccination against SARS-CoV-2 was actively offered to all subjects aged at least 12 years, while for children under 12 years old, the use of vaccine was still not approved. All the data have been collected by the Public Health Department of Modena Local Health Authority (AUSL Modena) through an application with a specific designed format for contact tracing in the pandemic period.

### 2.2. Data analysis

For continuous variables, we reported mean, standard deviation (SD), and range (min-max). For categorical variables, we reported absolute (N) and relative (%) frequencies. We performed the analyses in the entire study population and in selected subgroups. In particular, we subdivided the sample of teachers/students into not-vaccinated and vaccinated for SARS-CoV-2. This latter group was further divided according to the type of educational setting. To evaluate the spread of infection, secondary attack rate was calculated. Secondary attack rate was defined as the number of secondary cases exposed to index cases divided by total number of tested subjects exposed to index cases. We also compared the daily incidence of new cases over time occurred in the study sample with those occurred in the overall population of Modena province. Moreover, we assessed the influence of vaccination coverage on the SARS-CoV-2 spread of within schools using a logistic regression model adjusted for relevant confounders, sex, age group, type of educational setting and school role (teachers/students) for calculating the odds ratio (OR) with 95% confidential intervals (95% CI). Data analysis was performed using statistical software Stata v17.0 (StataCorp, College Station, TX, USA, 2021).

## 3. Results

From September 1 to December 15, 2021, 594 classes were followed by the Public Health Dept. of AUSL Modena due to the identification of an index case, with 13,934 subjects tested for SARS-CoV-2. Specifically, we included 1,400 teachers and 12,534 students within different educational settings, from nursery school up to high school. The identified clusters were 265 with a range of 2–22 total cases within the class (from 1 to 21 secondary cases). Through swab testing in the study population, 1,373 (9.9%) were identified as confirmed cases (10.6% of teachers and 9.8% of students), of whom 594 were classified as index cases and 779 as secondary cases. Among index cases, 101 (17.0%) were teachers and 493 (83.0%) were students; among secondary cases, 47 (6.0%) and 732 (94.0%), respectively.

We collected data on vaccination status. Within the study population, information on the vaccination coverage was missing for only 24 (0.2%) subjects, respectively 14 teachers and 10 students, and none of them was a confirmed case. These data are summarized in Table 1, along with socio-demographic characteristics.

**Table 1:** Socio-demographic characteristics and vaccination status of the total population (*n* = 13,934) and divided into different subgroups.

Characteristics	Male, N (%)	Female, N (%)	Age, mean ± SD (min-max)
Overall (*n* = 13,934)	6,545 (47.0)	7,389 (53.0)	13.9 ± 11.3 (0–68)
Teachers (*n* = 1,400)	209 (14.9)	1,191 (85.1)	43.7 ± 11.0 (19–68)
Students (*n* = 12,534)	6,336 (50.5)	6,198 (49.5)	10.5 ± 4.2 (0–22)
Index cases (*n* = 594)	282 (47.5)	312 (52.5)	15.8 ± 13.1 (0–63)
Secondary cases (*n* = 779)	370 (47.5)	409 (52.5)	11.1 ± 9.2 (1–65)
No infection (*n* = 12,561)	5,893 (46.9)	6,668 (53.1)	13.9 ± 11.3 (0–68)

Vaccination status		
	Yes N (%)	No N (%)
Overall (*n* = 13,910)	4,525 (32.5)	9,385 (67.5)
Teachers (*n* = 1,386)	895 (64.6)	491 (35.4)
Students (*n* = 12,524)	3,630 (29.0)	8,894 (71.0)
Students ≥12 years (*n* = 5,194)	3,630 (69.9)	1,564 (30.1)
Index cases (*n* = 594)	94 (15.8)	500 (84.2)
Secondary cases (*n* = 779)	61 (7.8)	718 (92.2)
Total cases (*n* = 1,373)	155 (11.3)	1,218 (88.7)
No infection (*n* = 12,537)	4,370 (34.9)	8,167 (65.1)
<12 years (*n* = 7,330)	–	7,330 (100.0)
≥12 years (*n* = 6,580)	4,525 (68.8)	2055 (31.2)

Information on the vaccination status was missing for 24 (0.2%) subjects, respectively 14 teachers and 10 students, and none of them were confirmed cases of SARS-CoV-2 infection.

Percentages are calculated excluding missing data on vaccination status.

Among all included subjects, 4,525 (32.5%) resulted to be vaccinated with at least one dose at the moment of exposure to virus. A higher percentage of vaccinated individuals (68.5%) could be observed considering only the population aged ≥12 years, for which the vaccination was regularly offered.

Out of 1,373 confirmed cases, only 155 (11.3%) were vaccinated, and the proportion decreased among secondary cases (7.8%), which are possibly related to school attendance.

In Tables 2, 3, we reported both secondary and index cases rate and secondary attack rate within the study population, divided into different subgroups based upon vaccination status or educational setting.

**Table 2:** Secondary/index cases rate and secondary attack rate within the study population, divided into different subgroups based on vaccination status.

	Total N	Index cases N (%)	Tested subjects following the index case N	Secondary cases N (%)**	No infection N (%)**	Secondary and index cases rate	Secondary attack rate
Vaccinated	4,525	94 (2.1)	4,431	61 (1.4)	4,370 (98.6)	0.65	1.4%
*Number of doses*							
1 dose	613	22 (3.6)	591	8 (1.4)	583 (98.6)	0.36	1.4%
2 doses*	3,804	72 (1.9)	3,732	52 (1.4)	3,680 (98.6)	0.72	1.4%
3 doses	108	-	108	1 (0.9)	107 (99.1)	-	0.9%
Non-vaccinated	9,385	500 (5.3)	8,885	718 (8.1)	8,167 (91.9)	1.44	8.1%
Overall	13,934	594 (4.3)	13,340	779 (5.8)	12,561 (94.2)	1.31	5.8%

Age groups							
<12 years	7,330	292 (4.0)	7,038	629 (8.9)	6,409 (91.1)	2.15	8.9%
≥12 years	6,604	302 (4.6)	6,302	150 (2.4)	6,152 (97.6)	0.50	2.4%
*Vaccination status*							
Vaccinated	4,525	94 (2.1)	4,431	61 (1.4)	4,370 (98.6)	0.65	1.4%
Non-vaccinated	2055	208 (10.1)	1847	89 (4.8)	1758 (95.2)	0.43	4.8%
Overall	13,934	594 (4.3)	13,340	779 (5.8)	12,561 (94.2)	1.31	5.8%

Percentages are calculated excluding missing data on vaccination status.

* Among those vaccinated with 2 doses, 48 are vaccinated > 6 months and 3,756 are vaccinated < 6 months.

** Percentages are calculated out of tested subjects.

**Table 3:** Secondary/index cases rate and secondary attack rate within study population, divided into different subgroups based on educational setting.

Type of educational setting		Total N	Index cases N	Tested subjects following the index case N	Secondary cases N	Secondary and index cases rate	Secondary attack rate
Nursery school (0–3 years)	Overall	420	27	393	10	0.37	2.5%
	Teachers	66	6	60	3		
	Students	354	21	333	7		
Infant school (3–5 years)	Overall	966	45	921	54	1.20	5.9%
	Teachers	138	18	120	7		
	Students	828	27	801	47		
Elementary school (6–10 years)	Overall	5,233	219	5,014	479	2.19	9.6%
	Teachers	622	37	585	29		
	Students	4,611	182	4,429	450		
Middle school (11–13 years)	Overall	3,732	154	3,578	176	1.14	4.9%
	Teachers	297	18	279	6		
	Students	3,435	136	3,299	170		
High school (14–19 years)	Overall	3,583	149	3,434	60	0.40	1.7%
	Teachers	277	22	255	2		
	Students	3,306	127	3,179	58		
Total	Overall	13,934	594	13,340	779	1.31	5.8%
	Teachers	1,400	101	1,299	47		
	Students	12,534	493	11,802	732		

Overall, the secondary attack rate for non-vaccinated subjects was higher than for vaccinated subjects (8.1% vs. 1.4%). A similar trend was also observed among people aged ≥12 years, who represented the target population for vaccination.

A logistic regression model was implemented to evaluate the vaccination coverage influence on the SARS-CoV-2 spread within schools. The results showed that being vaccinated was highly protective for risk of secondary infection within class following an index case (OR [95% CI]: 0.28 [0.20–0.40]). The analysis stratified by school role confirmed the protective effect of vaccination for students (OR [95% CI]: 0.41 [0.28–0.61]) and for teachers (OR [95% CI]: 0.09 [0.05–0.20]).

Higher secondary attack rates have been reported for subjects attending infant and elementary school (5.9 and 9.6%, respectively), compared to other types of educational settings. Indeed, lower secondary attack rates have been calculated for those who attend middle school and especially high school (4.9 and 1.7%, respectively). A low secondary attack rate (2.5%) was also seen for nursery school.

We also included data regarding the presence of symptoms in confirmed cases identified within our study population. As shown in Table 4, 662 (48.2%) confirmed subjects reported one or more symptoms, whereas 711 (51.8%) claimed to be asymptomatic. Among index cases, 391 (65.8%) subjects were symptomatic and 203 (34.2%) without symptoms. The proportion was inverted among secondary cases, 271 (34.8%) and 508 (65.2%), respectively.

**Table 4:** Presence of symptoms in confirmed cases identified within study population between September 1 and December 15.

	Symptom presence	
	**Yes N (%)**	No N (%)
*Index cases* *(n* *= 594)*	391 (65.8)	203 (34.2)
vaccinated (*n* = 94)	67 (71.3)	27 (28.7)
- 1 dose (*n* = 22)	13 (59.1)	9 (40.9)
- 2 doses *(*n* = 72)	54 (75.0)	18 (25.0)
non-vaccinated (*n* = 500)	324 (64.8)	176 (35.2)
*Secondary cases* (*n = 779*)	271 (34.8)	508 (65.2)
vaccinated (*n* = 61)	17 (27.9)	44 (72.1)
- 1 dose (*n* = 8)	2 (25.0)	6 (75.0)
- 2 doses*(*n* = 52)	15 (28.8)	37 (71.2)
- 3 doses (*n* = 1)	–	1 (100.0)
non-vaccinated (*n* = 718)	254 (35.4)	464 (64.6)
*Total cases* (*n* *= 1,373*)	662 (48.2)	711 (51.8)
vaccinated (*n* = 155)	84 (54.2)	71 (45.8)
- 1 dose (*n* = 30)	15 (50.0)	15 (50.0)
- 2 doses* (*n* = 124)	55 (44.4)	69 (55.6)
- 3 doses (*n* = 1)	–	1 (100.0)
non-vaccinated (*n* = 1,218)	578 (47.4)	640 (52.6)

Data are number (n) and percentage (%) for total cases (n = 1,373), and divided into different subgroups.

* Among those vaccinated with 2 doses, only 5 are vaccinated > 6 months and all those 5 subjects are symptomatic index cases.

**Figure 1:**
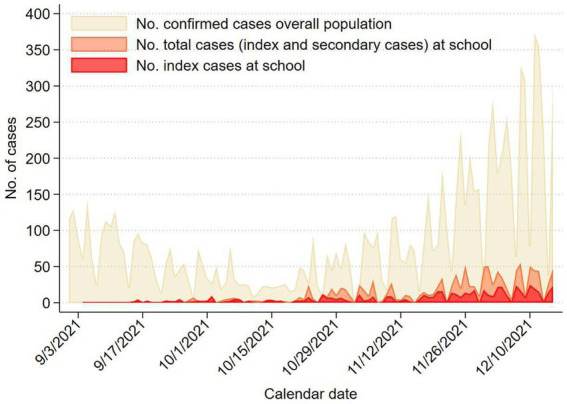
The daily number of confirmed cases in the overall population of Modena province, the number of total cases and of index cases in the scholastic population of Modena province from September 1 to December 15, 2021.

Figure 1 shows the total of daily cases and index cases at school, and confirmed cases in the entire population of the province of Modena. The trends nearly overlapped during our study period.

## 4. Discussion

This study aims to evaluate the spread of SARS-CoV-2 within schools of various types and grades in Modena province, from September to December 2021, further to rate the influence of vaccination in this environment.

The secondary attack rate was found higher in non-vaccinated subjects compared with value in vaccinated subjects (8.1% vs. 1.4%). Furthermore, the lowest secondary attack rate has been found for those who attended high school (1.7%).

Our results underline the effective protection of vaccination against SARS-CoV-2 as 88.7% of overall cases were not vaccinated. Particularly, the percentage of non-vaccinated subjects is 92.2% among secondary cases, which are those infections possibly related with school attendance. Therefore, vaccination shows a relevant impact in preventing transmission of SARS-CoV-2 within educational settings suggesting the collective beneficial effect of extensive vaccination in school population to reduce the outbreaks probability and the size (18). As a matter of that, comparison of secondary attack rates shows higher values for non-vaccinated subjects than vaccinated ones (8.1% vs. 1.4%). These data confirm that non-vaccinated subjects have a higher risk of contracting SARS-CoV-2 infection (33–35). As further demonstration for vaccination efficacy, lower secondary attack rate is reported for subjects with three doses of vaccine compared to those with only one dose (0.9% vs. 1.3%), suggesting that high levels of protection might be re-established through booster doses (36).

Based on different type of educational settings, lowest secondary attack rates were depicted for subjects attending middle school and especially high school (4.9 and 1.7%, respectively) compared to other settings. This difference may be explained by the observation that during the study period vaccination anti-SARS-CoV-2 was approved for people aged ≥12 years only. Our results show that having at least one vaccine dose is protective against transmission of virus, as reported by ECDC (20). In literature, widespread vaccine coverage is confirmed to be very important, especially among adolescents. Indeed, if the vaccine were not available to high school students, they would be expected to be at a higher risk of contracting the infection than primary school students, due to age-specific epidemiological characteristics and contact types (7).

It is interesting that a low secondary attack rate (2.5%) has been found also in nursery school. Possible explanations may be the different measures of isolation and quarantine adopted in that educational settings (32), the only one in which, during the study period, quarantine was disposed for all children of the same classroom, as a consequence of only one confirmed case. Moreover, analyzing transmission of respiratory disease in schoolchildren of different ages, in Japan Matsuda et al. have found a higher percentage of primary schools students with influenza than nursery or kindergarten children (23.4% *vs* 18.9%) over 5 influenza seasons (37).

It is also important to notice that many mitigation measures and health behavioral policies were adopted during our study period, according to ministerial and regional specific protocols (32): wearing a face mask was always mandatory, except for children aged <6 years and subjects with specific pathologies; periodic room ventilation and social distancing rules had to be guaranteed; the use of outdoor spaces was encouraged wherever possible.

Data regarding the symptoms presence in confirmed cases show a higher percentage of symptomatic individuals among index cases in comparison to secondary cases (65.8% vs. 34.8%). We can explain this difference due to the reason to perform swab testing within these two subgroups. Index cases were usually diagnosed with a positive swab test after the onset of symptoms. Instead, secondary cases underwent swab testing for screening protocol, as established by the Dept. of Public Health regardless of the symptoms presence (32).

According to our results, a comparison between scholastic cases and total cases in the overall population shows similar overlapping trends. This finding suggests that the SARS-CoV-2 transmission within schools was quite similar to the general virus circulation in Modena province over the same period; therefore, school contacts do not seem to have played a relevant role in the spread of the pandemic neither they have represented a higher risk factor for virus transmission, in line with the literature (12). Similarly, other studies revealed low rates of infection in school contacts, and SARS-CoV-2 circulation in schools was found to be much limited compared to the general population (17, 38).

A limitation of our investigations is that we did not have detailed data on symptoms but detailed information was not collected. However, considering the still limited evidence available on this topic, our findings expand the knowledge regarding the SARS-CoV-2 spread within schools and the impact of vaccination coverage in these settings. Some strengths should also be outlined. The epidemiological surveillance carried out by the Local Health Authorities were mandatory during the study period, thus occurrence of selection bias can be ruled out. Furthermore, the epidemiological investigation on the entire involved classes allowed a more in-depth study of transmission in different type of educational settings.

## 5. Conclusion

Vaccination against SARS-CoV-2 in children aged 12 years and older showed effectiveness in preventing virus transmission in school settings after the detection of an index case within their classroom. Indeed, a higher secondary attack rate was found among non-vaccinated subjects compared with vaccinated subjects. Furthermore, the lowest secondary attack rate has been found for those who attended high school. In conclusion, our findings highlight the importance of widespread anti-SARS-CoV-2 vaccination to reduce virus circulation also in school settings.

## Data availability statement

The datasets presented in this article are not readily available due to privacy and legal restrictions. Requests to access the datasets should be directed to the corresponding author.

## Ethics statement

The studies involving humans were approved by “Area Vasta Emilia Nord” Ethics Committee. The studies were conducted in accordance with the local legislation and institutional requirements. Written informed consent for participation in this study was provided by the participants and participants’ legal guardians/next of kin.

## Author contributions

SP, LB, and TF: conceptualization. SP and TF: methodology and funding acquisition. SP, MF, AD’A, and TF: formal analysis. SP, LB, AB, and TF: investigation. LI, AG, EF, GC, ZK, and SZ: resources and recruitment. SP, MF, LB, AD’A, LI, ZK, and SZ: data curation. SP, MF, and TF: writing—original draft preparation. SP, MF, LB, AD’A, LI, AG, EF, GC, ZK, SZ, AB, and TF: writing—review and editing. SP, TF, and AB: project administration. All authors have read and agreed to the published version of the manuscript.
